# Duodenal and cutaneous metastases from advanced rectal cancer: a rare case report

**DOI:** 10.3389/fonc.2026.1754861

**Published:** 2026-03-12

**Authors:** Jiaoling He, Yun Lin, Sikai Chen, Tingfang Zuo, Feilai Xie, Yan Zhang

**Affiliations:** 1 Fuzong Clinical Medical College of Fujian Medical University (900th Hospital of PLA Joint Logistic Support Force), Fuzhou, Fujian, China; 2 The School of Basic Medical Sciences, Fujian Medical University, Fuzhou, Fujian, China; 3 Fuzong Teaching Hospital of Fujian University of Traditional Chinese Medicine, Fuzhou, Fujian, China

**Keywords:** case report, cutaneous metastases, duodenal metastases, fruquintinib, rare, rectal cancer

## Abstract

Duodenal and cutaneous metastases from rectal cancer are rare and associated with poor prognosis. This case report describes a patient with advanced rectal cancer who developed these uncommon metastases after first-line therapy. Upon treatment with fruquintinib, the patient experienced a rapid and significant regression of the cutaneous lesions, demonstrating a visible clinical response. This suggests that fruquintinib may be an effective therapeutic option for patients with such aggressive and rare metastatic disease.

## Introduction

Rectal cancer is one of the leading causes of cancer-related deaths worldwide (1). In the metastatic setting, systemic therapy forms the cornerstone of treatment. The first-line standard typically involves combination chemotherapy (e.g., FOLFOX, FOLFIRI) alongside biologic agents targeting the vascular endothelial growth factor (VEGF) or epidermal growth factor receptor (EGFR) pathways, based on tumor molecular characteristics (2). Recent years have seen the integration of newer agents and regimens into clinical practice (3, 4). Despite these advances, disease progression following standard therapies remains universal, and distant metastasis is a leading cause of cancer-related mortality.

The liver and lungs are the most common sites of spread. In contrast, duodenal and cutaneous metastases are rare and associated with a poor prognosis, with a median time to death of 5.5 months (5). This underscores the unmet need for novel therapies in the refractory setting.

Fruquintinib is a highly selective oral inhibitor of vascular endothelial growth factor receptors (VEGFR) 1, 2, and 3. It was approved in China for the treatment of metastatic colorectal cancer (mCRC) refractory to standard chemotherapy in September 2018. This report presents a case of advanced rectal cancer with unusual metastatic sites that responded rapidly to fruquintinib.

## Case report

**Figure 1:**
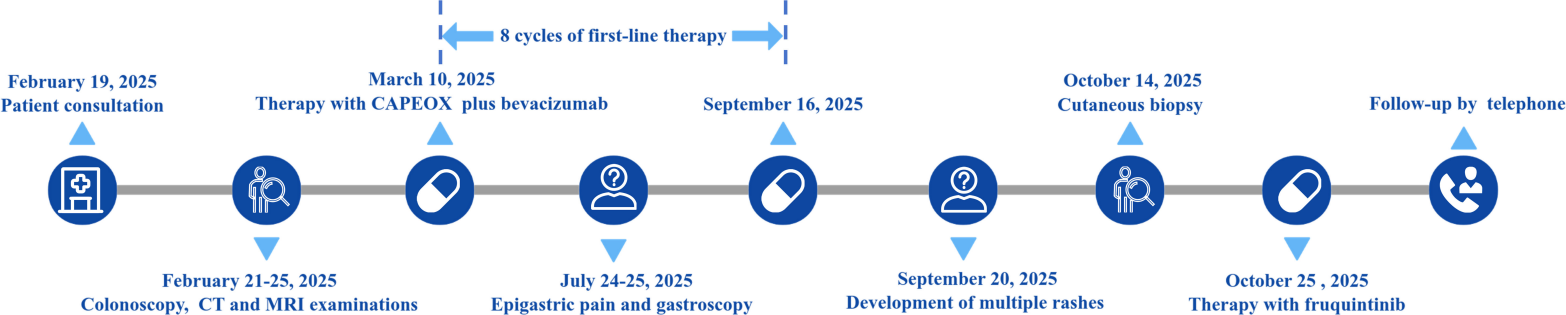
Timeline of the diagnosis and treatment process.

A 63-year-old male patient presented to our hospital (900th Hospital of PLA Joint Logistic Support Force) on February 19, 2025, with complaints of “increased frequency of bowel movements”. His ECOG (Eastern Cooperative Oncology Group) performance status (PS) was 1, with no significant prior medical or family history of cancer. Initial laboratory testings revealed elevated baseline tumor markers: carcinoembryonic antigen (CEA) at 10.61 ng/ml, carbohydrate antigen (CA) 12–5 at 56.20 U/ml and CA 19–9 at 525.80 U/ml. Subsequent colonoscopy identified a mass (5 × 3 cm) in the rectum, approximately 3.2 cm from the anal verge. The lesion exhibited an irregular surface with focal necrosis, was friable and bled easily on contact. Pathological examination confirmed an invasive moderately differentiated adenocarcinoma with molecular profiling showing mutation in NRAS exon 2 G12D/G12S, KRAS wild-type, BRAF wild-type, PIK3CA wild-type, proficient mismatch repair (pMMR), PD-L1 combined positive score (CPS) 20, and C-erbB-2 (1+). Given the patient’s financial constraints, the positron emission tomography/computed tomography (PET/CT) scan was omitted after multidisciplinary discussion. Computed tomography (CT) examination of the chest showed multiple lymph node metastases in the mediastinum, bilateral axillae and bilateral thoracic inlet regions. CT and magnetic resonance imaging (MRI) examinations of the total abdomen revealed a 4.5 × 3.9 cm mass in the left lobe of the liver, as well as multiple retroperitoneal lymph node metastases. No suspicious lymph nodes were identified in the mesorectal region. According to the Tumor, Node, Metastasis (TNM) staging system, the tumor was classified as stage IV B (T3N0M1b) per the National Comprehensive Cancer Network (NCCN) guidelines (2). Regarding therapeutic strategy, while a PD-L1 CPS of 20 suggested potential benefit from immune checkpoint inhibition, the pMMR status was the definitive negative predictive biomarker guiding the decision against immunotherapy. Moreover, the presence of an NRAS mutation precluded the use of anti-EGFR therapy (e.g., cetuximab). Thus, first-line therapy with CAPEOX (capecitabine and oxaliplatin) plus bevacizumab was initiated on March 10, 2025. The diagnostic and therapeutic timeline was summarized in **Figure 1**.

**Figure 2:**
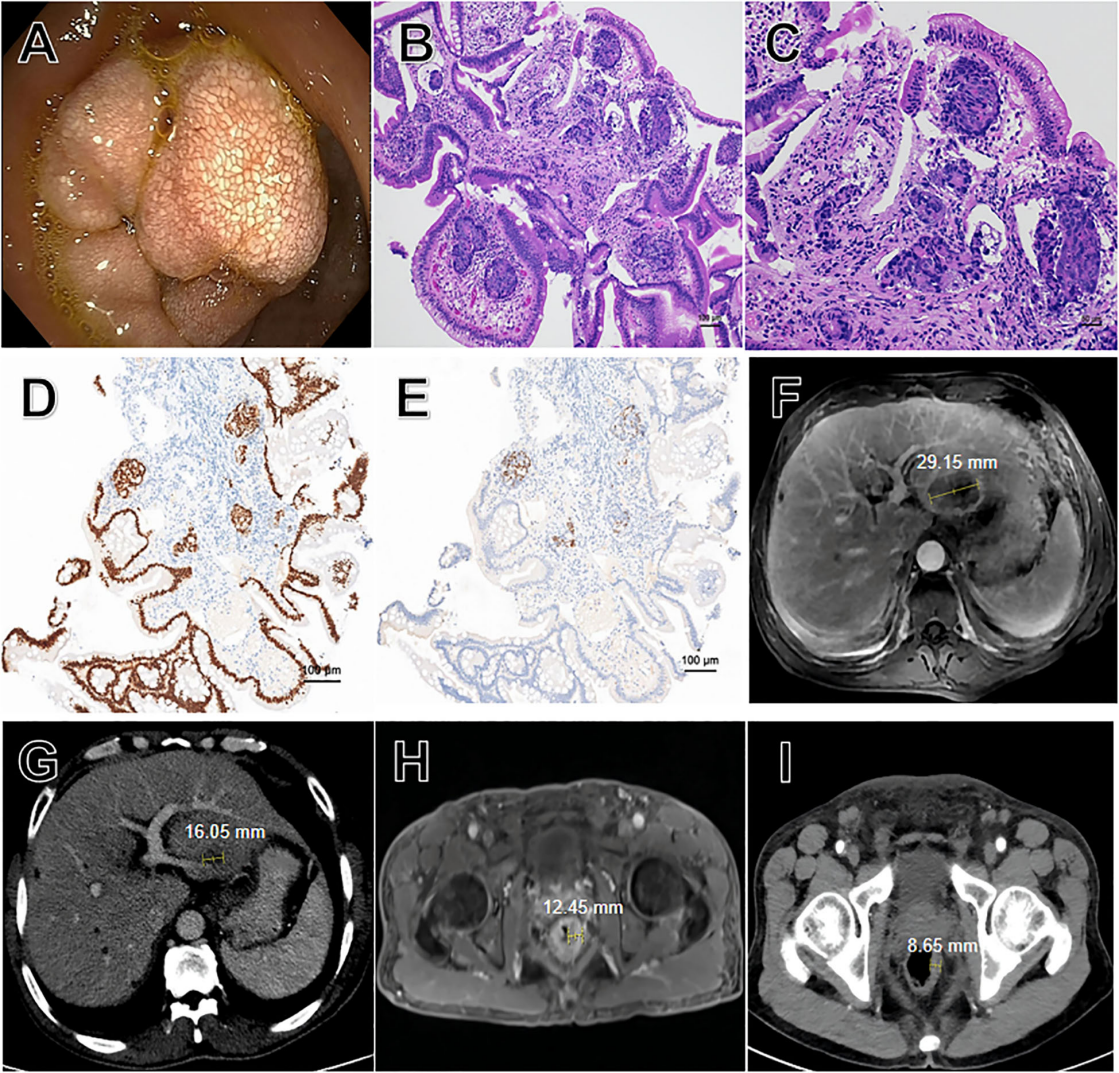
**(A–E)** for duodenal lesions and **(F–I)** for imaging of the liver and rectum. **(A)** Gastroscopic view of the duodenal bulb, revealing multiple masses with diffuse white punctate deposits. **(B)** Low-power view of the duodenal biopsy specimen, showing infiltrative nests of tumor cells within the mucosal stroma (Hematoxylin-Eosin staining, original magnification ×100). **(C)** Medium-power view of the duodenal biopsy specimen, demonstrating marked cytological atypia of tumor cells, including nuclear pleomorphism and hyperchromasia. Histological examination revealed moderately differentiated adenocarcinoma as a metastatic rectal cancer (Hematoxylin-Eosin staining, original magnification ×200). **(D)** Immunohistochemical staining demonstrated diffuse positivity for CDX-2 protein (original magnification ×100). **(E)** Immunohistochemical staining demonstrated diffuse positivity for SATB-2 protein (original magnification ×100). **(F)** MRI of the liver (July 3, 2025). **(G)** Enhanced CT scan of the liver (July 25, 2025). **(H)** MRI of the rectum (July 3, 2025). **(I)** Enhanced CT scan of the rectum (July 25, 2025).

After six cycles of the above therapy, the patient presented with epigastric pain on July 24, 2025. Gastroscopic biopsy identified metastatic lesions in the duodenal bulb (**Figures 2A–C**). Immunohistochemistry (IHC) showed positivity for CDX-2 and SATB-2 (**Figures 2D, E**); negativity for CK7; a Ki-67 index of 90%; and molecular profiling revealed an NRAS exon 2 mutation (G12D/G12S). We speculated these lesions were already present initially but went undetected due to the lack of PET/CT imaging. And follow-up MRI and CT scans respectively revealed a significant reduction in the size of the liver metastasis (45%, **Figures 2F, G**) and the rectal mass (31%, **Figures 2H, I**). Therefore, the overall response was assessed partial response (PR), and the first-line therapy was continued.

**Figure 3:**
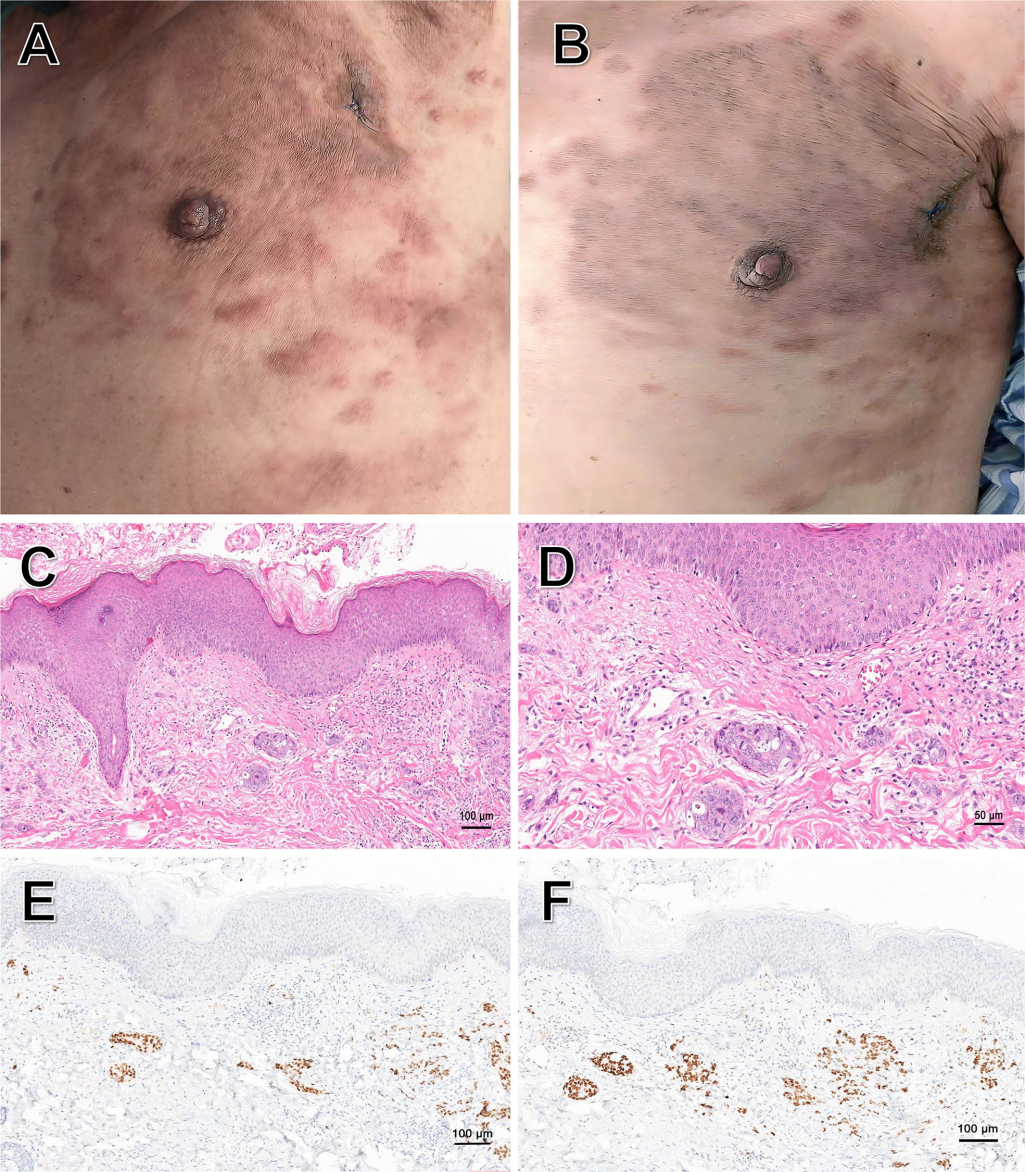
Cutaneous metastatic rashes and their histopathology. **(A)** Multiple red rashes on the left chest and epigastrium, with some areas coalescing into patches and exhibiting mild infiltration. **(B)** Rashes on the patient’s body on the seven day of oral fruquintinib. **(C)** Low-power view of the rash biopsy specimen, showing infiltrative nests of tumor cells in the dermis (Hematoxylin-Eosin staining, original magnification ×100). **(D)** Medium-power view of the rash biopsy specimen, demonstrating marked cytological atypia of tumor cells in the dermis, including nuclear pleomorphism and hyperchromasia. Histological examination revealed moderately differentiated adenocarcinoma as a metastatic rectal cancer (Hematoxylin-Eosin staining, original magnification ×200). **(E)** Immunohistochemical staining demonstrated diffuse positivity for CDX-2 protein (original magnification ×100). **(F)** Immunohistochemical staining demonstrated diffuse positivity for SATB-2 protein (original magnification ×100).

On September 20, 2025, the patient developed rashes on the left chest and epigastrium, which progressively increased in number (**Figure 3A**). On October 8, 2025, he came to our hospital for treatment. Based on the dermatologic morphology of firm, progressively enlarging nodules in the setting of widely metastatic disease, common drug eruptions (typically diffuse, pruritic, without a history of allergenic drug use), infections (typically painful, inflammatory and accompanied by fever) were considered less likely. Additionally, paraneoplastic dermatoses (which typically present with more inflammatory or characteristic patterns such as gyratum repens or dermatomyositis). Laboratory investigations revealed that the complete blood count and inflammatory markers (e.g., C-reactive protein, procalcitonin and eosinophil count) were within normal limits, and the autoantibody screen was negative. To definitively establish the diagnosis, a diagnostic excisional biopsy was performed.

Histopathological examination confirmed the presence of metastatic adenocarcinoma (**Figures 3C, D**). Immunohistochemical (IHC) staining (**Figures 3E, F**) exhibited co-expression of CDX-2 and SATB-2, absence of CK7, and a high Ki-67 index (90%). Subsequent molecular testing detected the same NRAS exon 2 mutation (G12D/G12S).

The emergence of new cutaneous metastases indicated progressive disease (PD). Given the patient’s deteriorated performance status (ECOG PS 3) and intolerance to standard second-line chemotherapy, third-line therapy was initiated directly. Based on the favorable overall survival (OS) and manageable safety profile reported in the FRESCO-2 trial (6), fruquintinib (5 mg, once a day, 3 weeks on/1 week off) was preferred over alternatives such as regorafenib or TAS-102 (7, 8). Notably, after seven days of treatment, a marked improvement in the cutaneous metastases was observed, characterized by decreased erythema, reduced size and diminished infiltration (**Figure 3B**). Laboratory studies revealed that CA19–9 decreased from 36.86 U/ml to 25.40 U/ml. The treatment was well-tolerated during hospitalization with no observed drug-related adverse events, and the patient was subsequently discharged.

Following discharge, the patient was transferred to a local hospital for continuing care, precluding the acquisition of new laboratory or imaging results. During several follow-up telephone calls, although advised to return to our hospital, the patient declined.

## Patient perspective

The patient provided informed consent for the publication of this case report. Our follow-up communications, primarily conducted via telephone, were focused on monitoring safety and adherence. During these communications, he reported continued adherence to fruquintinib without requiring dose adjustments. This suggests that the regimen was well-tolerated and manageable for him in an outpatient setting. He specifically denied experiencing adverse events such as hypertension, diarrhea or proteinuria.

## Discussion

Approximately 20% of individuals diagnosed with rectal cancer present with stage IV disease at initial diagnosis (9). The liver (73%), lungs (21%) and peritoneum are the most commonly observed sites of metastases (10). This report describes a patient with advanced rectal cancer who developed both duodenal and cutaneous metastases, with the latter showing a marked response to fruquintinib.

Primary malignancies originating from the small intestine are uncommon. The most frequent distinct histological types include neuroendocrine neoplasms (NENs), adenocarcinomas, lymphomas and gastrointestinal stromal tumors (GISTs) (11). Among these, the incidence of duodenal metastases is only 5% (11, 12). This rarity is reflected in recent case reports (13, 14). This case reinforces the duodenum as a rare but documented site of metastatic spread. The initial absence of PET/CT due to financial constraints precluded a definitive assessment of the metastatic timing. This highlights a critical challenge in resource-limited settings. However, PET/CT has inherent limitations, including high cost, substantial radiation and the potential for nonspecific findings (e.g., false positives from inflammation or false negatives in low-metabolic tumors) (15, 16). Therefore, a cost-effective and pragmatic initial staging approach may involve a combination of high-resolution CT or MRI of the head, chest, abdomen, and pelvis, complemented by bone scintigraphy and a thorough physical examination. This multimodal strategy, while potentially less sensitive than PET/CT for detecting micrometastases, provides a robust and clinically actionable assessment to guide initial therapy when comprehensive functional imaging is not feasible.

Similarly, cutaneous metastases are observed in approximately 0.6% to 10.4% of all cancer patients. They constitute about 2% of cases among all skin tumors (17) and 3.4% in colorectal cancer (18). Cutaneous metastases most commonly present as ulcerated dermal or subcutaneous nodules. They are characteristically firm, painless, and can be either single or multiple. Their presentation is occasionally nonspecific, mimicking benign entities including lipomas, neurofibromas and cellulitis (19). The appearance of cutaneous metastases is a recognized marker of aggressive disease biology and systemic dissemination, typically indicating a poor prognosis (18). Cutaneous metastases from rectal cancer most commonly occur in the inguinal and perianal regions, followed by the face, scalp and neck. Metastases to the chest and abdominal areas are relatively less frequent (20, 21). In this patient, their emergence signaled progression, but their dramatic regression within just seven days of starting fruquintinib provided a rapid, visible indicator of response. This suggests that easily monitorable cutaneous lesions can serve as valuable external biomarkers for the real-time assessment of therapeutic efficacy in advanced disease, underscoring the necessity of routine dermatological examination in these patients.

Cutaneous metastases are typically highly vascularized (22). Fruquintinib, as a highly selective VEGFR inhibitor, demonstrated potent anti-angiogenic effects, leading to rapid resolution. The rapid clinical response, manageable safety profile and oral administration position fruquintinib as a viable therapeutic alternative for patients with poor performance status.

The case is distinctive for the co-occurrence of two rare metastatic sites (duodenal and cutaneous) in a single patient, and for the strikingly rapid visual response of the cutaneous disease to fruquintinib. These features provide vivid clinical evidence of the drug’s antitumor activity. We believe studying such rare presentations is essential for personalizing late-line therapy. Future research should focus on molecular profiling of responsive lesions to understand their angiogenic and pathological basis.

Nevertheless, this is a single-case report with limitations, including a short follow-up period. The anecdotal visual regression, while compelling, requires confirmation in larger prospective studies to explore effective therapeutic strategies for patients with such rare metastases. Furthermore, the initial staging limitations due to financial constraints highlight a real-world challenge but also mean the precise timing and full extent of metastatic spread at diagnosis were not fully characterized.

## Data Availability

The original contributions presented in the study are included in the article. Further inquiries can be directed to the corresponding authors.
